# Biopsy of the olfactory epithelium from the superior nasal septum: is it possible to obtain neurons without damaging olfaction?

**DOI:** 10.1016/j.bjorl.2021.05.008

**Published:** 2021-06-05

**Authors:** Ellen Cristine Duarte Garcia, Lucas de Almeida Luz, Lucas Kanieski Anzolin, José Lucas Barbosa da Silva, Richard L. Doty, Fábio de Rezende Pinna, Richard Louis Voegels, Marco Aurélio Fornazieri

**Affiliations:** a Universidade Estadual de Londrina (UEL), Departamento de Cirurgia Clínica, Londrina, PR, Brazil; b University of Pennsylvania, Perelman Medicine School, Smell and Taste Center, Philadelphia, United States; c Universidade de São Paulo, Departamento de Otorrinolaringologia, São Paulo, SP, Brazil; d Pontifícia Universidade Católica do Paraná, Departamento de Medicina, Londrina, PR, Brazil

**Keywords:** Smell, Olfactory mucosa, Immunofluorescence, Biopsy, Neurons

## Abstract

**Introduction:**

Olfactory epithelium biopsy has been useful for studying diverse otorhinolaryngological and neurological diseases, including the potential to better understand the pathophysiology behind COVID-19 olfactory manifestations. However, the safety and efficacy of the technique for obtaining human olfactory epithelium are still not fully established.

**Objective:**

This study aimed to determine the safety and efficacy of harvesting olfactory epithelium cells, nerve bundles, and olfactory epithelium proper for morphological analysis from the superior nasal septum.

**Methods:**

During nasal surgery, 22 individuals without olfactory complaints underwent olfactory epithelium biopsies from the superior nasal septum. The efficacy of obtaining olfactory epithelium, verification of intact olfactory epithelium and the presence of nerve bundles in biopsies were assessed using immunofluorescence. Safety for the olfactory function was tested psychophysically using both unilateral and bilateral tests before and 1 month after the operative procedure.

**Results:**

Olfactory epithelium was found in 59.1% of the subjects. Of the samples, 50% were of the quality necessary for morphological characterization and 90.9% had nerve bundles. There was no difference in the psychophysical scores obtained in the bilateral olfactory test (University of Pennsylvania Smell Identification Test [UPSIT®]) between means before biopsy: 32.3 vs. postoperative: 32.5, *p* = 0.81. Also, no significant decrease occurred in unilateral testing (mean unilateral test scores 6 vs. 6.2, *p* = 0.46). None out of the 56 different odorant identification significantly diminished (*p* > 0.05).

**Conclusion:**

The technique depicted for olfactory epithelium biopsy is highly effective in obtaining neuronal olfactory tissue, but it has moderate efficacy in achieving samples useful for morphological analysis. Olfactory sensitivity remained intact.

## Highlights

• Olfactory biopsies from the superior part of the nasal septum did not significantly affect smell capacity.
• These biopsies obtain high rates of olfactory neurons.
• The described procedure also provides olfactory epithelium proper for morphological analysis.

## Introduction

Nasal mucosal biopsies play an important role in the investigation of the pathophysiology and potential treatment of a range of diseases. For example, biopsy studies of the olfactory epithelium (OE) can elucidate mechanisms of olfactory loss in chronic rhinosinusitis and post-infectious olfactory loss,^1^, ^2^ including that from viruses such as SARS-CoV-2,^3^, ^4^, ^5^ assist in the diagnosis and understanding of neurological diseases such as Alzheimer’s,^6^, ^7^ and enable innovative treatments with stem cells.^8^

The success rates of obtaining OE in biopsies performed from the nasal septum vary between 40% to 89%.^9^, ^10^, ^11^, ^12^, ^13^, ^14^, ^15^ However, these rates do not reveal the amount of intact OE that is harvested, a critical issue for morphological analyses, nor do they provide information about the percentage of samples that contain nerve bundles needed, for example, to culture neurons.

The identification of odors involves a complex afferent pathway with almost 400 olfactory receptors, each specific to certain odorous molecules alone or in combination.^16^, ^17^ In primates, the distribution of these receptors is not homogeneous throughout the nasal OE.^18^ Therefore, even a biopsy spanning a few millimeters could potentially generate disturbances in olfactory function. For this reason, it is important to evaluate whether biopsy procedures compromise the ability to smell any of a range of odorants. It was previously found that OE biopsies from the superior turbinate^19^ did not compromise subjects’ ability to smell any specific odorants. Since this region is near the cribriform plate and has the highest concentration of olfactory neurons,^20^ presumably it has significant redundancy so that any influences from an operative biopsy procedure would be expected to be minimal or non-existent. While the safety and efficacy of biopsies from nasal regions with less dense epithelia, such as the nasal septum, has been generally verified by others,^11^, ^21^ no studies have specifically sought to determine whether alterations to specific odorants or qualities occurs in such regions from this procedure.

The present study evaluated the efficacy rates of obtaining total OE, OE adequate for morphological analysis, and nerve bundles from the OE in superior nasal septum biopsies. We also sought to evaluate the safety of this procedure regarding the preservation of unilateral, bilateral, and specific olfactory function through standardized and validated olfactory tests, testing 56 different odors.

## Methods

### Study patients

This prospective cohort study enrolled 22 individuals awaiting nasal surgery due to nasal obstruction. They were non-smokers and had no olfactory complaints or histories of traumatic brain injury, neurodegenerative diseases, and chronic rhinosinusitis. During surgery, a biopsy of the olfactory mucosa from the superior nasal septum was performed unilaterally and the presence of OE confirmed through immunofluorescence. Safety was assessed bilaterally testing patients with the validated of the University of Pennsylvania Smell Identification Test (UPSIT).^22^, ^23^ The sixteen smells of the Sniffin’ Sticks identification part were used for the unilateral testing, eight odors for the right nasal cavity, and eight for the left, occluding the contralateral nostril with Microcopore®.^24^ Volunteers had their olfaction tested preoperatively and one month after surgery. The study was approved by the local ethics and research committee (CAAE: 41491014.1.0000.5231). Each participant or guardian signed the consent form after being aware of the methods and purposes of the study.

### Olfactory epithelium collection

**Figure 1:**
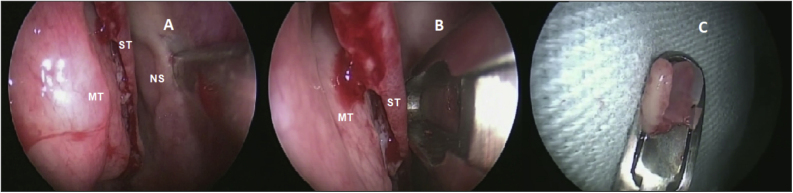
Biopsy performed on the superior nasal septum. (A) Detachment of the olfactory mucosa from the right nasal septum (NS) medial to the superior turbinate (ST) and medium turbinate (MT). (B) Cutting instrument removing the epithelium without damaging the tissue. (C) Olfactory epithelium harvested.

The same otolaryngologist collected the OE biopsies under general anesthesia. Without the use of pledgets to avoid damaging the epithelium, only three drops of oxymetazoline were applied inside the nostrils for local vasoconstriction. After the superior nasal septum mucosa detachment, a 3- to 5-mm diameter specimen was harvested with a cutting instrument (Fig. 1) from the lateral nasal septum medial to the superior turbinate. Only one biopsy was made per patient. Patients did not require local cauterization and the local bleeding spontaneously stopped.

### Immunofluorescence

The analysis of the presence and integrity of OE and neuronal bundles was performed using immunofluorescence. First, samples were fixed in 4% paraformaldehyde (PFA) for 24 h, then submerged in a 30% sucrose solution for the same period, and then in a mixture of 30% sucrose solution with Tissue-Tek O.C.T. compound (Sakura) for another 24 h. After that, the pieces were frozen in Tissue-Tek O.C.T. compound at −80 °C until analysis.

Tissues were cryosectioned at 15 μm (Leica CM 1520) and mounted on silanized slides (Jiangsu Huida Medical Instruments, China). Specimens were rinsed in 1× PBS (phosphate-saline buffer) in 3 washes of 5 min in a humid chamber. Afterward, antigen retrieval was performed on tubes with 2.16 mL of acetate buffer, 9.84 mL of citrate buffer, 108 mL of distilled water, and 60 μL of Tween 20, in a water bath at 90 °C. For thermal shock, the slides were placed on ice until they reached 25 °C. After that, the slides were deposited in a humid chamber and subjected to 3 washes with PBS 1× for 5 min each followed by antigen blocking with PBSt 0.1% solution and 3% BSA (bovine serum albumin). Soon thereafter, three more 5-min washes with PBS were made, and the slides were incubated overnight with primary anti-OMP (anti-Olfactory Marker Protein polyclonal antibody, Biorbyt) or anti-βIII (anti-beta tubulin III monoclonal antibody, Thermo Fisher), diluted 1:100 in 0.1% PBSt solution (Phosphate-Saline Buffer with triton) and 2% BSA. The biopsies were then washed three times with PBS 1× for 5 min and incubated with secondary antibody (anti-rabbit conjugated with DyLight® 650-GtxRb-003-D650NHSX 1:100 or anti-mouse conjugated with FITC - F2761 1:200) for 2 h. The slides were mounted using Fluoreguard Mounting Medium (SCYTEK). The sections were evaluated using a Leica Microsystems CMS TCS SP8 confocal microscope (Wetzlar, Germany). Negative controls were made from slides with tissue without the application of the primary antibody. The presence of OE was confirmed when the epithelium was marked by anti-OMP or anti-βIII. It was considered adequate for morphological characterization when at least 200 µm of OE with all cell layers was present. The presence of nerve bundles was evaluated in the lamina propria of the mucosa.

### Statistical analysis

Efficacy rates to obtain OE are described as percentages. Continuous variables, such as age and UPSIT scores, were expressed as means and standard deviations. Before and after the biopsy, olfactory tests scores were compared using the Student’s *t*-test after checking normality by the Shapiro-Wilk test. We made this comparison only in those patients who presented olfactory epithelium or nerve bundles in the collected samples (20 out of 22). Theoretically, patients without olfactory neurons would have no reason to exhibit decreased olfactory capacity. The level of significance was set at 0.05. Loss of capacity to identify specific odorants was performed with the 95% Confidence Intervals; if the intervals before and after the biopsy crossed, there was considered that no increase or diminishment of smell capacity occurred. A sample size of 8 participants pre- and post-biopsy would provide adequate power to reject the null hypothesis. We increased our sample in case of loss of followup. Sample size determination was based on a clinically meaningful difference of 4-points in the mean of the UPSIT scores, a power of 90%, a standard deviation of 6-points, and an alpha level of 5%.

## Results

**Figure 2:**
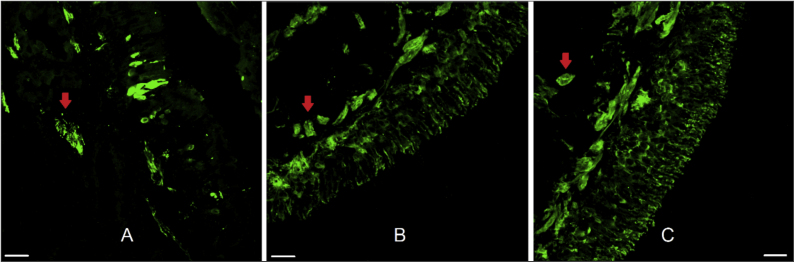
Human olfactory epithelium. Immunofluorescence of human olfactory epithelium suitable for morphological analysis in biopsy from the superior nasal septum with neuronal marking. In figure A we see anti-olfactory marker protein antibody (OMP 1:100, for mature olfactory neurons) marking. Figures B and C used the anti-beta tubulin III label (β 3 1:100, total, for mature and immature neurons). The red arrows indicate the presence of nerve bundles. Scale bar with 25 µm.

The twenty-two patients ranged in age from 14 to 58 years (mean = 31.1, SD = 12.5). Fifteen were men and seven were women. OE was obtained in 59.1% of the samples. Olfactory mucosa without tissue damage, proper for morphological analysis, was found in 50% of the samples and nerve bundles were observed in 90.9% (Fig. 2).

There was no difference in bilateral olfactory function before and after biopsy (mean UPSIT score before: 32.3 out of 40, SD: 1 vs. after: 32.5, SD = 1, *p* = 0.81). Six out of the 22 patients had a worse score on UPSIT after the biopsy, one by 1-point, three reduced by 2-points, the other two by three, one by 5-points, and the last by 8-points. Likewise, no significant differences were detected between the means of the unilateral olfactory test on the side where the biopsy was performed (mean before: 6 out of 8, SD = 0.3 vs. after: 6.2, SD = 0.3; *p* = 0.46) and on the contralateral nasal cavity (mean before: 5.7, SD = 0.4 vs. after: 6.2, SD = 0.3; *p* = 0.19). When comparing the variation in pre- and post-biopsy scores of the biopsied sides with the non-biopsied sides, there was no statistically significant difference (mean biopsied side: 0.2, SD = 0.2 vs. non-biopsied side: 0.5, SD = 0.4, *p* = 0.44).

The ability to identify each smell remained unimpaired on the bilateral (Table 1) and unilateral testing of both nasal cavities (Table 2, Table 3). Patients did not have any postoperative complications such as nasal bleeding, pain, infection, or cerebrospinal fluid (CSF) leak.

**Table 1:** Comparison of the percentage of correct answers by odor in the bilateral olfactory test before and one month after olfactory epithelium harvesting.

Odor	Before (% of correct answers, 95% CI)	After (% of correct answers, 95% CI)
Pizza	64.7 (37.7–84.7)	64.7 (37.7–84.8)
Bubble gum	76.5 (48.2–91.9)	70.6 (42.9–88.5)
Menthol	82.4 (53.7–94.9)	88.2 (59.2–97.5)
Cherry	82.4 (53.7–94.9)	88.2 (59.2–97.5)
Motor oil	70.6 (42.9–88.5)	52.9 (28.0–76.5)
Peppermint	94.1 (62.7–99.3)	94.1 (62.7–99.5)
Banana	82.4 (53.8–94.9)	58.8 (32.7–80.7)
Clove	94.1 (62.7–99.4)	88.2 (59.2–97.5)
Leather	100	100
Coconut	82.4 (53.5–94.9)	88.2 (59.2–97.5)
Onion	94.1 (62.7–99.4)	100
Fruit juice	88.2 (59.1–97.5)	94.1 (62.7–99.4)
Baby powder	94.1 (62.7–99.4)	100
Jasmine	64.7 (37.7–84.7)	70.6 (42.9–88.5)
Cinnamon	88.2 (59.1–97.5)	70.6 (42.9–88.5)
Gasoline	17.6 (5.1–46.3)	17.7 (5.1–46.3)
Strawberry	100	100
Coffee	64.7 (37.7–84.7)	47.1 (23.5–71.9)
Gingerbread	94.1 (62.7–99.3)	100
Apple	76.5 (48.2–91.9)	100
Perfume	94.1 (62.7–99.4)	82.4 (53.7–94.9)
Flower	76.5 (48.2–91.9)	88.2 (59.2–97.5)
Peach	82.4 (53.7–94.9)	100
Tire	94.1 (62.7–99.4)	88.2 (59.2–97.5)
Pickles	47.1 (23.5–71.9)	47.1 (23.5–71.9)
Pineapple	82.4 (53.7–94.3)	94.1 (62.7–99.4)
Raspberry	88.2 (59.2–97.5)	100
Orange	82.4 (53.7–94.9)	88.2 (59.2–97.5)
Walnuts	58.8 (32.7–80.7)	58.8 (32.7–80.7)
Watermelon	100	94.1 (62.7–99.4)
Solvent	82.4 (53.8–94.9)	76.5 (48.2–91.9)
Grass	70.6 (42.9–88.5)	64.7 (37.7–84.8)
Smoke	88.2 (59.1–97.5)	88.2 (59.2–97.5)
Wood	64.7 (37.7–84.8)	70.6 (42.3–88.5)
Grape	82.4 (53.7–94.9)	88.2 (59.2–97.5)
Garlic	94.1 (62.7–99.4)	88.2 (59.2–97.5)
Soap	94.1 (62.7–99.4)	88.2 (59.2–97.5)
Natural gas	94.1 (62.7–99.4)	94.1 (62.7–99.4)
Rose	58.8 (32.8–80.8)	52.9 (28.0–76.5)
Peanut	70.6 (42.9–88.5)	88.2 (59.1–97.5)

**Table 2:** Comparison of the percentage of correct answers by odor in the unilateral olfactory test before and one month after biopsy in the same nasal cavity of the biopsy.

Odor	Before (% of correct answers, 95% CI)	After (% of correct answers, 95% CI)
Orange	88.9 (37.4–99.1)	100
Leather	55.6 (19.5–86.6)	55.6 (19.5–86.6)
Cinnamon	44.5 (13.4–80.5)	66.7 (26.2–91.9)
Mint	100	100
Banana	88.9 (37.4–99.1)	88.9 (37.4–99.1)
Galician lemon	66.7 (26.2–91.9)	44.5 (13.4–80.5)
Licorice	44.5 (13.4–80.5)	55.6 (19.5–86.6)
Paint solvent	33.4 (8.1–73.8)	55.6 (19.5–86.6)
Garlic	100	87.5 (31.9–99.1)
Coffee	71.4 (21.5–95.8)	87.5 (31.9–99.1)
Apple	42.9 (9.1–84.9)	37.5 (8.7–79.2)
Clove	100	100
Pineapple	85.7 (25.7–99.1)	75.0 (27.3–95.9)
Rose	100	87.5 (31.9–99.1)
Fennel	100	100
Fish	100	100

**Table 3:** Comparison of the percentage of correct answers by odor in the unilateral olfactory test before and one month after biopsy on the contralateral nasal cavity.

Odor	Before (% of correct answers, 95% CI)	After (% of correct answers, 95% CI)
Orange	71.4 (21.5–95.8)	100
Leather	85.7 (25.7–99.1)	100
Cinnamon	85.7 (25.7–99.1)	50.0 (14.4–85.7)
Mint	100	100
Banana	100	100
Galician lemon	57.1 (15.1–90.9)	62.5 (20.8–91.4)
Licorice	71.4 (21.5–95.8)	75.0 (27.6–95.9)
Paint solvent	42.9 (9.1–84.9)	25.0 (4.1–72.4)
Garlix	88.9 (37.4–99.1)	88.9 (37.4–99.1)
Coffee	33.4 (8.1–73.8)	55.6 (19.5–86.6)
Apple	33.4 (8.1–73.8)	11.1 (0.9–62.6)
Clove	55.6 (19.5–86.6)	88.9 (37.4–99.1)
Pineapple	77.8 (32.9–96.1)	88.9 (37.4–99.1)
Rose	66.7 (26.2–91.9)	100
Fennel	88.9 (37.4–99.1)	88.9 (37.4–99.1)
Fish	88.9 (37.4–99.1)	100

## Discussion

This study showed that biopsies from the superior nasal septum did not meaningfully affect the patients’ olfactory sensitivity. Unilateral, bilateral function and the ability to identify each of the 56 odors tested was not compromised, confirming the safety of the technique. Importantly, the technique was highly effective in obtaining samples with nerve bundles, showing an excellent capacity for collecting olfactory neuronal tissue. A significant amount of mucosa adequate for morphological analysis was also obtained.

The safety of the OE biopsy from the superior septum was compatible with our previous study done with specimens from the superior turbinate.^19^ Our findings were also analogous to other studies that measured bilateral and unilateral smell ability after nasal septum biopsies to obtain OE.^11^, ^21^ Importantly, our research was the first to demonstrate the maintenance of individual smell capacities from each nasal cavity.

One could consider that the preservation of olfaction in our sample could result from the nasal function improvement after surgery, compensating a loss associated to the biopsy. Nevertheless, there is still controversy if nasal airway surgeries actually improve olfactory capacity. Using bilateral testing, an increase of olfactory test scores has already been found after this type of surgical procedure,^25^ especially in patients with worse olfaction. However, other authors depicted a diminishment in patients with higher preoperative scores.^26^ Interestingly, in our patients, when the identification capacities were tested on the nasal fossa contralateral to the biopsy, no difference was found in olfactory function. Thus, these data support that the nasal surgery per se does not affect this sense and confirms the security of harvesting the OE in the way that we describe.

Taking into account that we confirmed the presence of OE using immunofluorescence, we obtained 59.1% efficiency in obtaining OE from our biopies, with 50% of the specimens being of the quality needed for detailed morphological analysis. Previous studies harvesting OE from the superior septum had success in 40%–76% of the biopsies.^9^, ^12^, ^21^ When only immunohistochemistry is used, the rate of obtaining olfactory epithelium seems to be around 20% higher.^11^, ^19^ It is likely that the additional steps necessary for immunofluorescence processing results in compromising more the relatively small amount of tissue that is collected. This idea is supported by our finding of nerve bundles in most of the samples (90.9%) regardless of the presence of OE in many cases. Thus, we believe that the antigenic recovery technique, which aims to expose epitopes better and obtain a more defined marking,^15^, ^27^, ^28^ may have contributed to the loss of the epithelium during the procedure. Although this is a disadvantage of the immunofluorescence technique, it permits multiple markers at the same time, a characteristic immunohistochemistry cannot achieve. Finally, the high amount of nerve bundles indicates that the biopsy of this region is efficient in obtaining olfactory nerves, which is very useful for obtaining olfactory stem cells. An alternative to obtaining a better OE morphologic characterization would be to collect a larger amount of nasal mucosa, but this would potentially damage more this chemosensory function. Another possibility is to perform the biopsy in the upper part of superior turbinate. A previous collecting OE from this region showed that OE proper for morphological analysis was present in 62% of the specimens.^19^

As limitations, we did not include individuals at extreme ages (children or aged) and the small size of the specimen. For example, it is known that the older persons possess less OE than younger persons^29^ making this type of procedure potentially more harmful for olfactory function in these populations. Thus, the safety of the procedures described herein still needs to be assessed in these groups.

## Conclusions

Olfactory epithelium harvesting from the superior septum did not cause any olfactory disturbance when smelling with one or both nostrils. It also did not affect the capacity to identify the 56 individual odorants tested. The technique depicted effectively obtained neuronal olfactory tissue used for stem cells and had moderate effectiveness in providing samples without damage, adequate for morphological analysis.

## Conflicts of interest

The authors declare no conflicts of interest.

## Acknowledgements

Fundação Araucária de Apoio ao Desenvolvimento Científico e Tecnológico do Estado do Paraná (FA) and Coordenação de Aperfeiçoamento de Pessoal de Nível Superior (CAPES).
